# The motivation of breast cancer patients to participate in a national randomized control trial

**DOI:** 10.1007/s00520-023-07930-0

**Published:** 2023-07-15

**Authors:** C. Wegge-Larsen, M. Mehlsen, A. B. Jensen

**Affiliations:** 1 Department of Oncology, Aarhus University Hospital, Aarhus, Denmark; 2 Department of Psychology and Behavioral Sciences, Aarhus University, Aarhus, Denmark; 3 Institute of Clinical Medicine, Health, Aarhus University, Aarhus, Denmark

**Keywords:** Qualitative interview, Breast cancer, Motivation, Experience, Intervention-status, Personal resources

## Abstract

**Purpose:**

Clinical trials are essential for development of better cancer care. Therefore, patient willingness to participate in these trials is important. The aim of this study was to assess motivation and thoughts of breast cancer patients concerning participation in a clinical trial.

**Methods:**

Twenty-one patients participated in two semi-structed interviews about participating in a clinical trial testing the efficacy of cryotherapy for the prevention of chemotherapy-induced peripheral neuropathy in breast cancer patients treated with paclitaxel. The interviews took place before and after the intervention and were coded and categorized following the steps in Braun & Clarke’s thematic analysis to identify motivational factors and experiential themes.

**Results:**

Four overarching themes were identified: (1) reasons to participate in the trial, (2) personal resources, (3) safety, and (4) experience of the randomization. The most frequent reason for participating in the trial was to support research and help others, but many also participated hoping to receive the intervention treatment. The study showed that a surplus of personal resources played an important role when the patients decided to participate in the trial. Differences were found between patients belonging to the intervention and the control group in relation to these themes. Finally, both groups experienced the extra examinations received during the trial as an additional source of safety.

**Conclusion:**

This qualitative study found different factors influencing the experience of participating in a clinical trial, e.g., intervention-status, personal resources, and safety. This knowledge can be valuable when planning future clinical trials involving breast cancer patients.

## Introduction

Clinical trials are essential for evidence-based cancer care [1, 2]. A study by Meropol et al. showed that 84% of the patients were aware of clinical trials and thought clinical trials are important for improving cancer treatment [3]. However, other studies show that only 2 to 7 % of adult cancer patients agree to participate in clinical trials [4, 5]. Previous studies have investigated factors influencing decision-making process when patients choose to participate in clinical trials. An overview from 2020 described barriers and facilitators influencing patients’ decision-making related to clinical trial participation, identifying three types of influential facilitators: potential for personal benefit, altruism, and trust [6]. Another study investigating barriers and facilitators to trial participation also found that potential benefits of participation were important in patients’ decision-making as well as the opportunity to make a difference and help others [7]. A review by Bell et al. identified three overarching factors influencing decision-making related to trial participation among cancer patients: personal, social, and structural factors. Personal factors were age, disease stage, gender, ethnicity, and the patient’s knowledge and awareness of clinical trials. Social factors were defined as patients’ relationships in their social network (e.g., family and friends) and sociodemographic characteristics (e.g., education), while structural factors were trial logistics, e.g., travel distance to the study site and time needed for participating [8]. Keruakous et al. investigated common barriers for recruiting adult cancer patients in a qualitative study with research staff from cancer centers. They found that from the perspective of the research staff, the most commonly reported barriers were trial protocol-related issues, communication barriers and cultural beliefs, financial barriers, patients’ comorbidities, and physicians’ commitment [9]. Another barrier for taking part in clinical trials is that patients may feel like an experimental subject or a guinea pig [10]. Chatters et al. studied the experience of both participating in and running a randomized trial with patient in fertility treatment using qualitative interview with both staff and study participants. They found that study participants had positive preconceptions regarding the effect of the experimental treatment [11].

The literature points to several factors influencing trial participation in general, but more research is needed to investigate factors influencing participation specifically among breast cancer patients and how these patients later experience being part of a trial. Therefore, the aim of this study is to prospectively assess the motivation and thoughts concerning participation in a specific clinical trial among breast cancer patients, evaluating the effect of cryotherapy to reduce the risk of chemotherapy-induced peripheral neuropathy (CIPN) during treatment with paclitaxel, the CryoPac study [12]. CIPN is a large problem in treatment of breast cancer as it appears in 57–83 % of these patients [13–17]. The CryoPac study is a non-blinded study where the intervention group received cryotherapy with cooling gloves/socks. Participants in intervention and control group were treated in the same room. Therefore, everyone knew who were in the control group and who were in the intervention group and had the opportunity to discuss this while receiving chemotherapy. We wanted to investigate if this influenced participants motivation to continue their participation in the trial.

## Methods

### Study population and recruitment

Participants were recruited from the CryoPac study sample. All participants in the CryoPac study were examined with a neurophysiological examination before and after treatment with paclitaxel. In connection to the first neurophysiological examination, performed by the first author, twenty-one patients were invited to participate in two semi-structured interviews about their motivation and experience concerning participation in the CryoPac study. The study took place at a university hospital with a high research activity, so most participants were informed about several clinical trials.

### Procedure

The participants were recruited to this study right after randomization in the CryoPac study, in connection with the first neurophysiological evaluation. The first interview took place immediately after inclusion and contained questions about their motivation to participate in the trial. The second interview took place at either the 8th treatment with paclitaxel or in connection with the second neurophysiological evaluation after end of treatment. The second interview contained questions about the participants’ experience of being part of the clinical trial and their motivation to continue participating in the trial. Both interviews were performed as semi-structured interviews, using an interview guide, and in both interviews, participants were asked to rank their motivation to participate on a scale from 1 to 10. Hand-written notes were taken in connection to the first interview, while the second more comprehensive interview was audio recorded and transcribed verbatim.

Two out of the 21 participants in the interview dropped out of the CryoPac trial during the paclitaxel treatment period; still, both agreed to participate in the second interview. Here, they were asked about the reason for their dropout and their experience of participating in the trial.

### Data analysis

The qualitative data were coded and categorized following the steps in Braun & Clarke’s thematic analysis to identify motivational factors and experiential themes. Braun & Clarke’s framework for thematic analysis consists of six phases: familiarizing with the data, initial coding, searching for themes, reviewing themes, defining themes, and producing the report [18]. After transcription and familiarizing with the data by reading and re-reading the interviews, the initial coding of the data set began. From this initial coding, a list of different codes was identified, which were then sorted into potential themes and subthemes. Themes and subthemes were reviewed and refined, and the final overarching themes and subthemes related to the overall aim of the study were found. Data from the interviews were then analyzed using the overarching themes and subthemes and described.

Furthermore, an average was calculated for their motivation score for participating and their motivation to continue participating in the study. These two scores were compared to evaluate if the motivation for participation changed over time and if the two randomization groups differed in their motivation to continue participation in the CryoPac trial.

## Results

All 21 participants included in the study completed both interviews; twelve participants were from the intervention group and nine from the control group. Two of the participants dropped out of the CryoPac trial during the study period, one from each of the two groups. The motivation score for participating, respectively, continuing in the clinical trial is presented in Table 1. Participants who continued in the trial were equally or more motivated at the second interview, when comparing their first and second motivation scores.

**Table 1:** Age and motivation score of the participants

Randomization	Age	Motivation for entering the trial (score 1–10) 1st interview	Motivation for continued trial participation (score 1–10) 2nd interview
Intervention group	Average: 50 (Range: 32–62)	Average: 9.4 (Range: 8–10)	Average: 9.8 (Range: 8–10)
Control group	Average: 51 (Range: 41–68)	Average: 8 (Range: 6–10)	Average: 8.9 (Range: 7–10)

In the qualitative analysis, four overarching themes were identified in the two interviews: (1) reasons to participate in the clinical trial, (2) personal resources, (3) safety, and (4) experience of the randomization.

### Reasons to participate in the clinical trial

Four topics were mostly mentioned by the participants, when asked about their reason to participate in the trial: support research, altruism, getting the intervention treatment, and fear of late complications (Table 2). Twelve participants mentioned that they agreed to participate in the clinical trial to support research. An equal number of participants wanted to participate in the trial to help others (altruism). Four participants mentioned a combination of supporting research and helping others as the reason to participate in the trial. Additionally, ten participants also wanted to take part in the trial to get the intervention treatment. Most of these participants wanted to participate in the clinical trial to support research or help others in addition to receiving the intervention treatment. Six patients mentioned that they accepted to participate in the trial because they had a fear of developing late complications. Six of the participants only mentioned one reason why they agreed to participate. Eleven patients mentioned two reasons why they wanted to participate, while the last four mentioned three reasons why they wanted to take part in the clinical trial. In the intervention group, the main reason to participate was to help others (altruism), which 83 % mentioned. In the control group, the main reasons to participate were to support research and to get the intervention treatment, mentioned by 67 %.

**Table 2:** Reasons to participate in the clinical trial as mentioned by the participants

Themes Condensation *Exemplifying quotes*	
Support research The reason to participate in the trial was to support research “It is the only way we can grow wiser. So, I agreed to participate to help science on its way.” *– Participant 17 (control group)*	
Altruism The reason to participate in the clinical trial was to help other future patients “I agreed to participate for the sake of future patients, so you can get knowledge that can improve their treatment.” *– Participant 13 (intervention group)*	
Support research and Altruism The reason to participate in the clinical trial were a combination of helping other future patients and supporting research “To help others in the long run (…) I want to support research, so we can grow wiser, which can help future patients.” *– Participant 14 (intervention group)*	
To get the intervention treatment The reason to participate in the clinical trial was to get the intervention treatment “I wanted to participate in the clinical trial to get the cooling treatment because I wanted to reduce the risk of getting sensory disturbances.” *– Participant 5 (control group)*	
Fear of late complications The reason to participate in the clinical trial was because of a fear of late complications and a hope that the intervention treatment could reduce these late complications “Additionally, I agreed to participate in the clinical trial because I worry about late complications. That is why I will do what I can to avoid these. I still got lots of years left and that is why I worry about late complications.” – *Participant 19 (intervention group)*	

### Personal resources

Another theme, which was prominent in the participants’ considerations about taking part in the clinical trial, was that of personal resources (Table 3). This theme relates to a need for a surplus of mental energy for patients to accept participation in the clinical trial. Five of the participants thought about the comprehensiveness of a clinical trial, e.g., how long the study would last, how much time they needed to spend filling out questionnaires, and how well they were informed about the study, which were seen as important factors when deciding to take part in the trial. Additionally, three participants mentioned that the number of clinical trials they were informed about also affects whether they had the surplus of personal resources to take part in clinical trials, because most patients do not have the personal resources to take part in all suggested clinical trials. A participant mentioned that some patients may be excluded from clinical trials due to the personal and economic costs of participating. Two participants stated that it was important for them to know that they had the opportunity to leave the study, if they could not manage study participation. Twelve participants thought that taking part in a clinical trial demands personal resources. Factors that influence their decision whether to take part in the trial were, for instance, being aptly informed and the opportunity to back out. Factors seen as barrier for taking part in the trial were personal and economic costs and the feeling of being introduced to an overwhelming number of clinical trials.

**Table 3:** It demands personal resources to agree to participate in a clinical trial

Themes Condensation *Exemplifying quotes*	
Personal resources This theme relates to the required need for a surplus of mental strength and capacity for agreeing to participate in the clinical trial “I think it is a psychological thing whether you agree or not.” – *Participant 6 (control group)*	
The comprehensiveness of the clinical trial The comprehensiveness of a clinical trial refers to how long the study participation is and if the study is easy to understand “I think that the more long-run the study is, the more I think ‘I cannot handle this’. So long projects where you will be contacted again in 2 years, I decline to participate in.” *– Participant 10 (control group)* “It is always important when you are offered to participate that you understand what it is all about, basically what do you want to study. I think that is important, so you know what is going on.” *– Participant 9 (intervention group)*	
Many projects Some of the participants have had considerations regarding the number of clinical trials they were offered to participate in as they have had to decline some due to their lack of energy “I think more about the number of clinical trials you are introduced to when you get the message that you have cancer. Then I thought ‘well, this is fine, I will say “yes” to this and “no” to something else because it becomes too much’. I don’t know how many women will say “yes” to every clinical trial, there’s probably some who do. (…) So, I think it is more about the extent to which you have to participate when you cannot manage it in the first place.” *– Participant 7 (intervention group)*	
Personal and economic costs One participant mentioned that some patients may be excluded from the clinical trials due to the personal and economic costs of participating “I have a surplus of mental and physical resources but there is a lot of patients who struggle in everyday life because the chemo is tough and they don’t have any energy, so I would imagine that some do not have the strength to participate this time [the neurophysiological examination]. So, it would be a shame and it is not only this clinical trial, but it is probably also like that in a lot of other clinical trials, and I think it is a shame that some lose their interest because all their resources are drawn upon both economically and personally.” – *Participant 12 (control group)*	
Opportunity to back out of the study The opportunity to back out of the study played an important role when deciding to participate in the clinical trial “It was also good to know that if I could not handle it, I could back out. It was also a good thing that you know that you do not agree to participate and then you must finish, there is always an opportunity to back out again.” – *Participant 1 (intervention group)*	

### Safety

In relation to participants’ concerns about safety, two subthemes emerged: extra examinations and discomfort (Table 4). Most participants mentioned that they were pleased with the extra examinations in the trial and that the examination made them feel safer. Many participants also mentioned that they found it positive that we could schedule the extra examinations on days they already had another appointment at the hospital, so they did not have to come to the hospital once more. One participant from the control group felt that being in the control group created discomfort, because she was dissatisfied with the fact that all patients was not offered the intervention treatment. On the other hand, seven participants from the intervention group felt discomfort by receiving the intervention treatment. This caused two participants in the trial to discontinue their cooling treatment. Eight participants mentioned discomfort related to not being able to use their hands during the cooling treatment. Some mentioned that presenting new participants to the cooling treatment could demystify the cooling treatment so that more patients would agree to participate in the trial.

**Table 4:** The theme about safety consists of the subthemes extra examination and discomfort with being in the control group and discomfort from the intervention treatment

Themes Condensation *Exemplifying quotes*	
Extra examinations Many participants mentioned that they were pleased with the extra examination that was performed in the clinical trial and they felt great safety by having the examination “(…) I think it is really nice that you from the beginning get an examination of your nerves and subsequently I know where I am, and I also know that in a year. I think that is incredibly positive, because one thing is what I am feeling, and another thing is what is being measured.” *–Participant 11 (intervention group)* “It was pleasant that it (the neurophysiological examination) could take place in addition to other treatments I have had, so that has been nice (…). It was pleasant that you did not have to come out her for an extra visit.” *– Participant 20 (control group)*	
Discomfort with being in the control group This subtheme refers to a participant who was annoyed of being part of the control group “I have thought that we in the control group kind of were guinea pigs and that was not a pleasant feeling. Maybe that is also because you can find so much information saying that it works. So, it seems a bit foolish that you are not just giving the cooling treatment. It is hard being the one who is not receiving the cooling treatment.” *– Participant 2 (control group)*	
Discomfort from the intervention treatment Some mentioned that receiving the intervention treatment created discomfort because the cooling treatment was uncomfortable and because they could not use their hands because of the cooling gloves “I just felt that I could not be bothered with having more pain than necessary. It felt like my fingers were about to break or fall of. It hurts so much.” *– Participant 9 (intervention group)* “And then there is the thing with you not being able to do anything, you are sitting locked. You cannot use your hands, that is tough.” *– Participant 16 (intervention group)* “Maybe if some patients were doubtful, it would be a good idea to have a cooling-glove which people could try on. (…) So that in the conversation you could take a pair of cooling-gloves with you so that you could try it and feel what it is, how does it look and how does it feel, so you could see that it is not that bad.” *– Participant 1 (intervention group)*	

### Experience of the randomization

In relation to the participants’ experience of the randomization process, there was a difference between the two randomization groups (Table 5). Six participants from the control group were annoyed in the beginning that they have not been offered the intervention treatment. Two of these six felt they were cheated, because they wished they have been offered the intervention treatment. The majority from the control group who were annoyed in the beginning later accepted being in the control group. Two participants mentioned that because they did not develop neuropathy, it was okay that they were not offered the cooling treatment. Two others accepted being in the control group because they rationalized that they could not know if it would have made any difference concerning their neuropathy if they had been randomized to the intervention, since some participants in the intervention group also developed neuropathy. Some participants accepted being in the control group because they heard that the intervention treatment could create discomfort. A subtheme observed equally frequent in the two groups were curiosity. Participants from the control group were curious about whether the cooling treatment worked and had asked the participants from the intervention group about this. The participants from the intervention group on the other hand had experienced other patients being curious about the cooling treatment, and it was often a topic of conversation when they received the intervention treatment. Five participants from the intervention group were happy that they were randomized to the cooling treatment when they were together with other patients who did not receive the cooling treatment. One of the participants from the intervention group mentions having felt more privileged when she was sitting next to participants from the control group. Additionally, five of the participants from the intervention group had worries about side effects from the intervention when they were sitting next to patients from the control group.

**Table 5:** The experience of the randomization

Themes Condensation *Exemplifying quotes*	
Felt cheated being in the control group Some participants from the control group felt cheated because not being offered the intervention treatment “I have often been sitting together with other participants who have worn the cooling-gloves. In this situation, I have sometimes felt a bit cheated because I have thought that I would also like to do that.” *– Participant 2 (control group)*	
Accepted being in the control group Many of the participants from the control group were annoyed in the beginning that they were not randomized to the cooling treatment, but they later accepted being in the control group “If after 3-4 times I had thought ‘now I can feel something, damn it, I wish I have been randomized to the intervention treatment’ (…). But luckily, I have not had any sensory disturbances. So, it was okay that I was not in the active part of the study.” *– Participant 8 (Control group)* “I have heard from others who have been treated with the cooling treatment, who had a good effect of it. So, I wanted to get the cooling treatment, but now I know that there might also be side effects to the cooling treatment, so I am fine with being in the control group.” *– Participant 18 (control group)* “Of course, I have thought about whether I could have avoided it if I had received it, but when I then hear from the ones, I know who have had it on but still have some of the same symptoms, then I think that there wasn’t a big difference.” – *Participant 5 (control group)*	
Curiosity The subtheme about curiosity were mentioned in both groups. Participants from the control group had been curious about if the cooling treatment had an effect. Participants from the intervention group have experienced other patients being curious about the cooling treatment “I asked about it. Some must take them off because it is too cold and uncomfortable. So, I have asked if they have any sensory disturbances, and the ones I have talked to, haven’t had any.” *– Participant 12 (control group)* “We have actually talked a lot about it. It has often been a topic of conversation, because they have asked why and because I have been sitting with it (the cooling treatment).” *– Participant 21 (intervention group)*	
Feeling more privileged A participant from the intervention group felt more privileged when she has been sitting next to participants from the control group “There was someone who had her own pair of cooling-gloves with her because she was not chosen to the cooling treatment, so she had brought her own. In that situation, I felt more privileged you can say than she was.” – *Participant 4 (intervention group)*	
Side effects to the cooling treatment Some of the participants have experienced side effects to the cooling treatment “(…) And then I have been thinking about if the other participants think it as uncomfortable as I think it is.” *– Participant 15 (intervention group)* “It is like a straitjacket. You cannot do anything at all.” *– Participant 4 (intervention group)*	

Two participants dropped out of the CryoPac study during the study period. The participant from the intervention group dropped out because of side effects to the cooling treatment. The participant from the control group dropped out because she purchased cooling gloves for herself. Despite the difference between the two groups regarding the experience of randomization to the control group or the intervention group, it is important to note that all 21 participants wanted to recommend others to take part in the clinical trial.

## Discussion

We found that the most common reasons to participate in the clinical trial were to support research and help others. Another frequent reason to participate were to receive the cooling treatment. A study by Sheridan et al. found that the potential for personal benefit from the study was the most commonly reported facilitator of research participation [6]. In the present study, ten participants mentioned in the interview that they agreed to participate in the clinical trial to get the cooling treatment, even though they were aware of the randomization procedure deciding if they got the cooling treatment. Out of those, nine also wanted to participate in the clinical trial to support research or help others. This indicates that even though many participants in the clinical trial were motivated by the benefit of getting the cooling treatment, most also wanted to help others and support research. Our findings so confirm the findings in a review by Bell et al. that the desire to help others (altruism) are a predictor for participating in clinical trials [8].

Another major theme in the interviews focused on how taking part in a clinical trial demand personal resources. The participants mentioned factors that influence the decision to take part in the clinical trial, such as being informed, the comprehensiveness of the clinical trial, and the opportunity to back out of the trial. Barriers for taking part in clinical trials were personal and financial costs by participating and the large number of clinical trials introduced to patients. The latter meaning, the sheer number of trials they were presented to had a negative effect on the mental resources of the participants. An important finding, as these patients could be presented for several trials in the beginning of their breast cancer treatment.

Many of the participants mentioned that the extra examination in the clinical trial induced a felling of safety. They felt their treatment was better when participating in the trial because of the additional follow-ups. However, one participant mentioned that some patients may not have energy to take part in extra examinations because of lack of energy that the cancer and the treatment induces. A study by Keruakous et al. found that frequent laboratory testing and office visits were a barrier to the patients’ willingness to participate in clinical trials [9]. This indicates that extra examination can also be seen a barrier to take part in a clinical trial, because it demands more energy from the participants. In the CryoPac study, the staff tried to schedule extra examination on days where the participants were already at the hospital for other appointments, and this may explain why participants in this study regarded the extra examination as a benefit and not a nuisance. The type of examination may also affect whether extra examinations are seen as barriers or facilitators to take part in a clinical trial, e.g., patients might not find an extra blood sample useful for themselves, while an examination of nerve function can be seen as useful.

One of the participants mentioned that she experienced discomfort due to being in the control group and did not understand why everyone was not just given the cooling treatment. A study by Chatters et al. found that participants often had positive preconceptions regarding the effect of the intervention treatment. They also found that patients randomized for the control group felt disappointed and struggled to understand the need for a control arm in the study [11]. This indicates that the outcome of the randomization can play an important role in the participants’ decision to continue participation in a trial. On the other hand, we found that even though most participants from the control group were disappointed due to their group allocation in the beginning of the study, they later accepted being in the control group and stayed in the trial. This suggests that even though the outcome of the randomization plays an important role in the beginning of a study because of positive preconceptions regarding the effects of the intervention, participants may later accept the outcome of the randomization, maybe because most participants also agreed to participate due to more altruistic reasons.

Two of the participants from the intervention group had to stop receiving the intervention treatment because of side effects, emphasizing that receiving the intervention treatment can also create discomfort in a clinical trial.

In the intervention group, the main reason to participate in the study was to help others, while in the control group, the main reason to participate was to receive the intervention treatment and further to support research. Although a slightly lower motivation for staying in the trial was observed in the control group compared to the intervention group, this did not transform into a higher dropout rate. Regarding the theme of personal resources, more participants from the control group mentioned that it demands a surplus of personal resources to take part in a clinical trial. This might be because the participants from the control group did not receive the intervention treatment as they wished for and therefore have thought more about the costs of participating in the trial. Regarding the theme of safety, there was no substantial difference between the two groups.

Methodologically, a strength of this study was that all patients asked to participate in the study, agreed to participate. Another strength is that the study also contains interviews with participants that decided to drop out of the CryoPac study during the study period. Additionally, it is a strength that the participants agreed to be take part in two interviews, an interview before starting treatment and a more comprehensive interview after paclitaxel treatment, making it possible to follow up on the motivation to participate in the clinical trial and be informed about their experience with participating in the trial throughout the study period.

A limitation in this study was that the participants were aware of which randomization group they belonged to when they answered the questions about their motivation to participate and the reason they decided to participate in the trial. This could have affected the participants’ answers. Additionally, a further limitation of the study is that only participants who decided to take part in the clinical trial were interviewed and consequently we do not have information from patients who declined to take part in the CryoPac study. To further understand the motivation to participate in randomized trials this study could have benefitted from having interviews with participants declined participating in the trial.

The results from this qualitative study may be applicable when planning future clinical trial. Some practical implications are that it is important to plan the clinical trial such that it is easy to understand for the participants. The personal resources of the participants are also important to consider when informing patients about new clinical trials, so it does not become too overwhelming when they are asked to participate in different clinical trials and to engage in extra examinations due to the trial participation. Extra examinations in clinical trials can be experienced as an advantage by the patients, which can be used when informing new participants about a clinical trial. Finally, healthcare staff may be informed that initial disappointment of being allocated to the control group is reduced over time in most patients.

## Conclusion

This qualitative study found that the most common reasons to participate in the clinical trial were to support research and help others. The second most common reason to participate was to receive the intervention treatment. Having a sufficient surplus of personal resources were found to be an important factor when deciding to take part in a clinical trial. Extra examination was found to induce a feeling of safety. Some participants were initially displeased with participating, because they did not receive the cooling treatment, but later accepted being in the control group. Knowledge of factors influencing the experience of participating in a clinical trial such as intervention-status, personal resources, and safety can be valuable for planning future clinical trials.
